# Frequency of *EGFR* T790M mutation and multimutational profiles of rebiopsy samples from non-small cell lung cancer developing acquired resistance to EGFR tyrosine kinase inhibitors in Japanese patients

**DOI:** 10.1186/s12885-016-2902-0

**Published:** 2016-11-08

**Authors:** Ryo Ko, Hirotsugu Kenmotsu, Masakuni Serizawa, Yasuhiro Koh, Kazushige Wakuda, Akira Ono, Tetsuhiko Taira, Tateaki Naito, Haruyasu Murakami, Mitsuhiro Isaka, Masahiro Endo, Takashi Nakajima, Yasuhisa Ohde, Nobuyuki Yamamoto, Kazuhisa Takahashi, Toshiaki Takahashi

**Affiliations:** 1 Division of Thoracic Oncology, Shizuoka Cancer Center, 1007 Shimonagakubo, Nagaizumi-cho, Sunto-gun, Shizuoka 411-8777 Japan; 2 Department of Respiratory Medicine, Juntendo University Graduate School of Medicine, 2-1-1 Hongo, Bunkyo-ku, Tokyo 113-8421 Japan; 3 Division of Drug Discovery and Development, Shizuoka Cancer Center Research Institute, 1007 Shimonagakubo, Nagaizumi-cho, Sunto-gun, Shizuoka 411-8777 Japan; 4 Third Department of Internal Medicine, Wakayama Medical University, 811-1 Kimiidera, Wakayama, 641-8509 Japan; 5 Division of Thoracic Surgery, Shizuoka Cancer Center, 1007 Shimonagakubo, Nagaizumi-cho, Sunto-gun, Shizuoka 411-8777 Japan; 6 Division of Diagnostic Radiology, Shizuoka Cancer Center, 1007 Shimonagakubo, Nagaizumi-cho, Sunto-gun, Shizuoka 411-8777 Japan; 7 Division of Diagnostic Pathology, Shizuoka Cancer Center, 1007 Shimonagakubo, Nagaizumi-cho, Sunto-gun, Shizuoka 411-8777 Japan

**Keywords:** Non-small cell lung cancer, Epidermal growth factor receptor mutation, Rebiopsy, T790M mutation

## Abstract

**Background:**

The majority of non-small cell lung cancer (NSCLC) patients with epidermal growth factor receptor (*EGFR*) mutation eventually develop resistance to EGFR tyrosine kinase inhibitors (TKIs). Minimal information exists regarding genetic alterations in rebiopsy samples from Asian NSCLC patients who develop acquired resistance to EGFR-TKIs.

**Methods:**

We retrospectively reviewed the medical records of patients with NSCLC harboring *EGFR* mutations who had undergone rebiopsies after developing acquired resistance to EGFR-TKIs. We analyzed 27 practicable samples using a tumor genotyping panel to assess 23 hot-spot sites of genetic alterations in nine genes (*EGFR*, *KRAS*, *BRAF*, *PIK3CA*, *NRAS*, *MEK1*, *AKT1*, *PTEN*, and *HER2*), gene copy number of *EGFR*, *MET*, *PIK3CA*, *FGFR1*, and *FGFR2*, and *ALK*, *ROS1*, and *RET* fusions. Additionally, 34 samples were analyzed by commercially available *EGFR* mutation tests.

**Results:**

Sixty-one patients underwent rebiopsy. Twenty-seven samples were analyzed using our tumor genotyping panel, and 34 samples were analyzed for *EGFR* mutations only by commercial clinical laboratories. Twenty-one patients (34 %) had *EGFR* T790M mutation. Using our tumor genotyping panel, *MET* gene copy number gain was observed in two of 27 (7 %) samples. Twenty patients received continuous treatment with EGFR-TKIs even after disease progression, and 11 of these patients had T790M mutation in rebiopsy samples. In contrast, only 10 of 41 patients who finished EGFR-TKI treatment at disease progression had T790M mutation. The frequency of T790M mutation in patients who received continuous treatment with EGFR-TKIs after disease progression was significantly higher than that in patients who finished EGFR-TKI treatment at disease progression (55 % versus 24 %, *p* = 0.018).

**Conclusions:**

The frequency of T790M mutation in this study was lower than that in previous reports examining western patients. These results suggest that continuous treatment with EGFR-TKI after disease progression may enhance the frequency of *EGFR* T790M mutation in rebiopsy samples.

**Electronic supplementary material:**

The online version of this article (doi:10.1186/s12885-016-2902-0) contains supplementary material, which is available to authorized users.

## Background

Lung cancer is the most common cause of cancer-related deaths, and non-small cell lung cancer (NSCLC) accounts for approximately 85 % of all lung cancers [1, 2]. Over 70 % of patients with NSCLC have advanced disease at the time of diagnosis, and prognosis is generally poor [3]. Recently, molecular targeted therapies have been developed and have provided a remarkable benefit to NSCLC patients with specific genetic alterations. In particular, NSCLC with mutation in the epidermal growth factor receptor (EGFR) gene are sensitive to EGFR blockade with specific tyrosine kinase inhibitors (TKIs). EGFR-TKIs are efficacious in patients with NSCLC harboring *EGFR* mutations as demonstrated in prospective clinical trials [4–8]. However, in spite of this efficacy almost all patients with *EGFR*-mutant NSCLC develop resistance to EGFR-TKIs.

Various mechanisms of resistance to EGFR-TKIs have been identified, and understanding these is critical for development of effective treatment strategies for EGFR-TKI-resistant NSCLC. The major mechanism of acquired resistance reported is secondary T790M mutation on exon 20 on the *EGFR* gene [9–12]. This secondary mutation enhances ATP-binding affinity of *EGFR*-mutated cells. Since EGFR-TKIs are competitive ATP-inhibitors, their efficacy is decreased in the face of the T790M mutation [13]. Additional mechanisms include amplification of the *MET* gene [11, 12, 14], *PIK3CA* mutation [11, 15], *BRAF* mutation [16], epithelial-to-mesenchymal transition (EMT) [11], and small cell lung cancer (SCLC) transformation [11, 12].

Several studies have examined the mechanisms and frequency of EGFR-TKI resistance, though minimal data regarding Japanese patients exist. Furthermore, the clinical factors that influence the frequency of acquired resistance mutations, especially T790M, remain unclear. This study aimed to analyze the causes of acquired resistance to EGFR-TKIs in Japanese patients with NSCLC, and to evaluate clinical factors related the frequency of T790M mutation.

## Methods

### Patients

We reviewed the medical records of consecutive patients with NSCLC harboring *EGFR* mutations who had undergone rebiopsies based on physician’s decision in the cases of acquired resistance to EGFR-TKI. Most rebiopsy samples were obtained from sites assessed as disease progression by imaging. Patients were treated at the Shizuoka Cancer Center between September 2002 and August 2014. Acquired resistance was defined according to Jackman’s criteria [17]. The criteria defined acquired resistance as progression while receiving EGFR-TKI, after initial response or durable stable disease (>6 months). The written informed consent regarding *EGFR* mutational analysis was obtained from most patients, and verbal informed was from some patients since *EGFR* mutational analysis was performed under the Japanese insurance system. Additionally, some patients were enrolled in the Shizuoka Lung Cancer Mutation Study [18], and these samples were analyzed using our tumor genotyping panel. This study protocol was approved by the Institutional Review Board of Shizuoka Cancer Center under number 27–J102–27–1–3.

### Mutational profiling

A tumor genotyping panel was designed to assess 23 hotspot sites of genetic alterations in 9 genes (*EGFR*, *KRAS*, *BRAF*, *PIK3CA*, *NRAS*, *MEK1*, *AKT1*, *PTEN*, and *HER2*), gene copy number of *EGFR*, *MET*, *PIK3CA*, *FGFR1*, and *FGFR2*, and *ALK*, *ROS1*, and *RET* fusions using pyrosequencing plus capillary electrophoresis, quantitative polymerase chain reaction (PCR), and reverse transcription PCR, respectively (Table 1). We analyzed samples from patients enrolled in the Shizuoka Lung Cancer Mutation Study, using this tumor genotyping panel. The other samples were analyzed for *EGFR* mutations using the Scorpion ARMS or Cycleave methods by a commercial clinical laboratory (SRL Inc., Tokyo, Japan) (see Additional file 1).

**Table 1:** Multiplexed tumor genotyping panel

Gene name	Position	AA mutant	Nucleotide mutant
EGFR	G719	G719	2155G > T/A
		G719A	2156G > C
	exon 19	Deletion	
	T790	T790M	2369C > T
	exon20	Insertion	
	L858	L858R	2573 T > G
	L861	L861Q	2582 T > A
KRAS	G12	G12C/S/R	34G > T/A/C
		G12V/A/D	35G > T/C/A
	G13	G13C/S/R	37G > T/A/C
		G13D/A	38G > A/C
	Q61	Q61K	181C > A
		Q61R/L	182A > G/T
		Q61H	183A > T/C
BRAF	G466	G466V	1397G > T
	G469	G469A	1406G > C
	L597	L597V	1789C > G
	V600	V600E	1799 T > A
PIK3CA	E542	E542K	1624G > A
	E545	E545K/Q	1633G > A/C
	H1047	H1047R	3140A > G
NRAS	Q61	Q61K	181C > A
		Q61L/R	182A > T/G
MEK1 (MAP2K1)	Q56	Q56P	167A > C
	K57	K57N	171G > T
	D67	D67N	199G > A
AKT1	E17	E17K	49G > A
PTEN	R233	R233	697C > T
HER2	exon20	Insertion	

### Evaluation of efficacy

Responsiveness to EGFR-TKI treatment was evaluated according to the Response Evaluation Criteria in Solid Tumors version 1.1 [19]. Progression-free survival (PFS) was defined as the period between the start of EGFR-TKI treatment and progressive disease or death from any cause. Overall survival (OS) was defined as the period between the start of EGFR-TKI treatment and the date of death from any cause.

### Statistical analysis

All categorical variables were analyzed by the chi-square test or Fisher’s exact test, as appropriate. Continuous variables were analyzed using the Mann-Whitney test. Logistic regression analyses were used to adjust for potential confounding factors. All *p* values < 0.05 were considered statistically significant. All analyses were performed using JMP 10 for Windows statistical software (SAS Institute Japan Inc., Tokyo, Japan).

## Results

### Patient characteristics

Sixty-one patients with NSCLC harboring *EGFR* mutations, and who had undergone rebiopsy after acquired resistance to EGFR-TKI at the Shizuoka Cancer Center were included in this study. Patient characteristics are shown in Table 2. The median age (range) was 64 (39–84) years, and most patients were female (72 %) and never-smokers. All patients had been diagnosed with adenocarcinoma of the lung with activating *EGFR* mutations at initial diagnosis. The types of *EGFR* mutations before the initial EGFR-TKI treatment were exon 19 deletion in 37 patients (61 %), exon 21 L858R in 19 patients (31 %), and other/double *EGFR* mutations in five patients (8 %). Thirty-nine patients (64 %) were treated with EGFR-TKI as first-line therapy. Twenty-two patients (36 %) received EGFR-TKI as second or subsequent-line therapy. Forty-nine patients (80 %) were treated with gefitinib, seven patients (12 %) with erlotinib, and five patients (8 %) with other EGFR-TKIs including afatinib. All patients received EGFR-TKI monotherapy. Twenty patients received continuous treatment with EGFR-TKI more than 30 days after disease progression, and 41 patients finished EGFR-TKI treatment within 29 days after diagnosis of disease progression.

**Table 2:** Patient characteristics analyzed in our study (*n* = 61)

Age, year	
Median	64
Range	39–84
Sex, n (%)	
Female	44 (72 %)
Male	17 (28 %)
Smoking history, n (%)	
Never	44 (72 %)
Former/Current	17 (28 %)
ECOG performance status, n (%)	
0–1	52 (85 %)
2–4	9 (15 %)
Pretreatment EGFR status, n (%)	
Exon19 deletion	37 (61 %)
Exon21 L858R	19 (31 %)
Other	5 (8 %)
EGFR TKI, n (%)	
Gefitinib	49 (80 %)
Erlotinib	7 (12 %)
2nd generation	5 (8 %)

Abbreviations: *ECOG* eastern cooperative oncology group, *EGFR* epidermal growth factor receptor, *TKI* tyrosine kinase inhibitor

### Rebiopsy

Table 3 depicts characteristics of rebiopsy sites, specimens, and procedures in patients who had undergone rebiopsy after developing acquired resistance to EGFR-TKIs. Because of their easy accessibility and practical necessity, serous effusions such as pleural effusion and cerebrospinal fluid account for more than half of the specimens. Pulmonary lesions were also rebiopsied, with the most common procedure being transbronchial biopsy. Biopsy samples from lymph nodes or other sites were obtained using computed tomography-guided or sonography-guided needle biopsy. All rebiopsies were performed after stopping EGFR-TKI treatment.

**Table 3:** Procedures and specimens of rebiopsy samples obtained from NSCLC patients with *EGFR* mutations

Procedure and specimen	Number
Surgery	
Brain	3
Lung	2
Autopsy	1
Biopsy	
Lung	15
Lymph node	3
Other	4
Fluid	
Pleural effusion	24
Cerebrospinal fluid	8
Cardiac effusion	1

### Resistance mechanisms

**Fig. 1:**
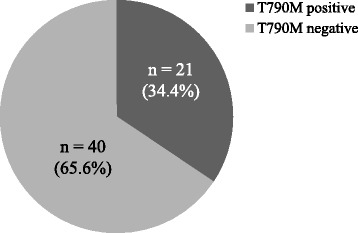
Frequency of T790M mutation in rebiopsy samples (*n* = 61)

**Fig. 2:**
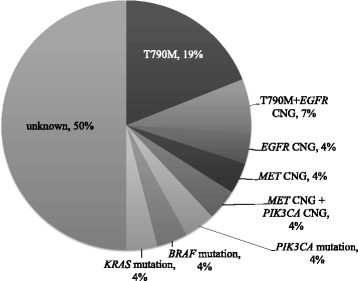
Multimutational profiling in rebiopsy samples analyzed using our tumor genotyping panel (*n* = 27). CNG: Copy number gain

A total of 61 rebiopsy samples were analyzed for EGFR mutations. Twenty-seven rebiopsy samples were analyzed using our tumor genotyping panel, and 34 samples were examined for *EGFR* mutations by commercial clinical laboratories. All of 61 patients had *EGFR* activating mutations before EGFR-TKI treatment, and 55 patients (90.2 %) still had same *EGFR* mutations in rebiopsy samples. T790M mutation was identified in 21 of 61 samples (34.4 %; Fig. 1). No samples had small cell histologic transformation. In samples analyzed using our tumor genotyping panel, *MET* gene copy number gain was seen in two of 27 samples (7 %). Additionally, we detected *PIK3CA* mutation (E542K), *BRAF* mutation (G466V), and *KRAS* mutation (G12D), in one sample each in 27 samples (4 %) (Fig. 2). Six of 61 rebiopsy samples (9.8 %) did not possess *EGFR* mutation, despite having EGFR activating mutations at the initial analysis. *KRAS* mutation was detected in 1 of these samples.

### T790M prevalence

**Fig. 3:**
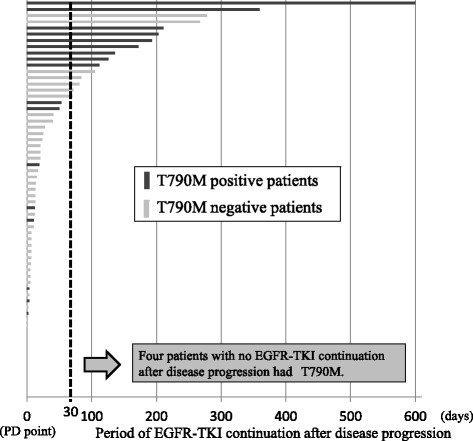
Relationship of EGFR-TKI continuation beyond progressive disease and T790M prevalence

Correlations between patient characteristics and T790M prevalence were evaluated (Table 4). Eleven of 20 patients who received continuous treatment with EGFR-TKI after disease progression had T790M mutation in the rebiopsy sample. However, only 10 of 41 patients who had finished EGFR-TKI treatment at the time of disease progression had T790M mutation (Fig. 3). The frequency of T790M mutation in patients who received continued treatment with EGFR-TKI after disease progression was significantly higher than in patients who finished EGFR-TKI at diagnosis of disease progression (55 % versus 24 %, *p* = 0.018). Multivariate analysis also demonstrated that continuous treatment with EGFR-TKI after disease progression was significantly correlated with T790M mutation (Table 4). Other characteristics, including PFS with EGFR-TKI, rebiopsy site, and rebiopsy sample, had no statistical association with the prevalence of T790M.

**Table 4:** Multivariate and univariate analyses of patient characteristics and T790M prevalence in patients with NSCLC harboring *EGFR* mutations, who had undergone rebiopsy after acquired resistance to EGFR-TKI (*n* = 61)

Patient characteristics	Number	T790M (%)	*P* (Univariate)	*P* (Multivariate)
Age			0.9292	
≥75	12	4 (33 %)		
<74	49	17 (35 %)		
Sex			0.4904	
Female	44	14 (32 %)		
Male	17	7 (41 %)		
Smoking history			0.4904	
Never	44	14 (32 %)		
Former/current	17	7 (41 %)		
EGFR mutation status			0.1038	
Exon19 deletion	37	9 (24 %)		
Exon21 L858R	19	9 (47 %)		
Other	5	3 (60 %)		
Rebiopsy site			0.5813	0.9133
Central nervous system	11	3 (27 %)		
Other	50	18 (36 %)		
Rebiopsy sample			0.2017	0.5016
Tissue	28	12 (43 %)		
Fluid	33	9 (27 %)		
EGFR TKI			0.1208	
Gefitinib	49	17 (35 %)		
Erlotinib	7	4 (57 %)		
2nd generation	5	0 (0 %)		
Line of EGFR-TKI			0.4235	
1st	39	12 (31 %)		
2nd or later	22	9 (41 %)		
History of platinum doublet until rebiopsy			0.7021	
Yes	34	11 (32 %)		
No	27	10 (37 %)		
PFS with EGFR-TKI			0.4823	
≥10 months	34	13 (38 %)		
<10 months	27	8 (30 %)		
Interval between RECIST PD and rebiopsy			0.2766	
≥4 months	29	12 (41 %)		
<4 months	32	9 (28 %)		
Period of continuation of TKI beyond PD			0.0182	0.0417
≥30 days	20	11 (55 %)		
<30 days	41	10 (24 %)		

Abbreviations: *EGFR* epidermal growth factor receptor, *TKI* tyrosine kinase inhibitor, *PFS* progression free survival, *PD* progressive disease

## Discussion

Previous reports from examining patients in western countries have reported *EGFR* T790M mutation in 49–69 % patients with NSCLC harboring *EGFR* mutations who had undergone rebiopsy after developing acquired resistance to EGFR-TKIs [11, 12, 20]. In contrast, our study identified T790M mutation in only 21 of 61 rebiopsy samples (34.4 %). This finding is similar to that of the one other Japanese study we are aware of [21]. Therefore, T790M prevalence in Japanese and Western patients may be different. In our study, only 30 % of patients received continuous treatment with EGFR-TKI after disease progression. Shimilarly, few such patients were included in the study from Hata et al. [21]. However, 88–91 % of patients in previous studies from western countries received continuous treatment with EGFR-TKI after disease progression [12, 20]. Additionally, the frequency of T790M mutation in patients who received continuous treatment with EGFR-TKI after disease progression was significantly higher than that in patients who had finished EGFR-TKI treatment by diagnosis of disease progression in our study. Furthermore, the preclinical report showed that continuous exposure to EGFR-TKIs induced T790M mutation in a NSCLC cell line with an *EGFR*-sensitive mutation [22]. These data suggest that continued treatment with EGFR-TKIs after disease progression may promote T790M mutation. While differences in ethnicity and analysis methods may underlie these inconsistencies, the potential for EGFR-TKIs to promote T790M mutation should not be overlooked.

The frequencies of *MET* gene copy number gain and *PIK3CA* mutation in our study were similar to those previously reported in studies from western countries [11, 12]. Furthermore, *BRAF* mutation is associated with acquired resistance to EGFR-TKIs [16]. We also detected *KRAS* mutation in one rebiopsy sample. *KRAS* and *EGFR* mutations have previously been considered mutually exclusive [23]. However, Kuiper et al. recently reported *KRAS* mutation in one rebiopsy sample following development of acquired resistance to EGFR-TKIs [24]. Furthermore, Li et al. have identified double mutation of *EGFR* and *KRAS* in pretreatment assessment of NSCLC patients [25]. These data suggest that *KRAS* mutation may promote acquired resistance to EGFR-TKIs through drug selective pressure. However, more data are required to confirm this hypothesis.

The availability of continuous treatment with EGFR-TKIs after disease progression is still controversial. In IMPRESS trial, continuation of gefitinib treatment after disease progression on gefitinib monotherapy did not prolong progression-free survival and overall survival in patients who received platinum-based doublet chemotherapy as subsequent line of treatment [26]. However, it is unclear that the efficacy of continuous using EGFR-TKIs without platinum doublets [27, 28]. Recently, we had been able to use third generation EGFR-TKIs that have great efficacy for NSCLC with *EGFR* T790M mutation in clinical practice. If there are relationship between the continuous treatment with EGFR-TKIs after disease progression and the frequency of T790M, the continuous therapy can be more important choice.

Our study had several limitations. First, we retrospectively collected the data from a single institution, and our sample size was small. This small sample size results from the difficulty surrounding rebiopsy in clinical practice. Second, we analyzed only 27 rebiopsy samples (44.3 %) using our tumor genotyping panel. Therefore, further multi-institutional studies are warranted to verify our results.

## Conclusions

The frequency of T790M mutation in rebiopsy samples in our study was lower than that reported in previous reports studies of western patients. The frequency of T790M mutation in patients who received continuous treatment with EGFR-TKIs after disease progression was significantly higher than that in patients who stopped EGFR-TKI treatment at diagnosis of disease progression. Continuous treatment with EGFR-TKI following disease progression may therefore influence the frequency of *EGFR* T790M mutations in rebiopsy samples.

## Additional file

Additional file 1: The detail of mutational analysis. (DOCX 22 kb)

## Abbreviations

EGFR: Epidermal growth factor receptor
EMT: Epithelial-to-mesenchymal transition
NSCLC: Non-small cell lung cancer
OS: Overall survival
PCR: Polymerase chain reaction
PFS: Progression-free survival
SCLC: Small cell lung cancer
TKI: Tyrosine kinase inhibitor
