# Supplementary material for: Frequency of EGFR T790M mutation and multimutational profiles of rebiopsy samples from non-small cell lung cancer developing acquired resistance to EGFR tyrosine kinase inhibitors in Japanese patients
Source: BMC Cancer. 2016 Nov 8;16:864. doi: 10.1186/s12885-016-2902-0 (PMC5100094; doi:10.1186/s12885-016-2902-0)
Supplement: Additional file 1: — The detail of mutational analysis. (DOCX 22 kb) [file 12885_2016_2902_MOESM1_ESM.docx]

**Frequency of EGFR T790M mutation and mulimutational profiles of rebiopsy samples from non-small cell lung cancer developing acquired resistance to EGFR tyrosine kinase inhibitors in Japanese patients.**

**Ko *et al.***

**Supplemental file**

1. **The tumor genotyping panel in the Shizuoka Lung Cancer Mutation Study**

*Nucleic acid sample preparation*

Genomic DNAs were extracted from surgically resected tissues and/or tumor biopsies using QIAamp DNA mini kit (QIAGEN, Hilden, Germany). QIAamp DNA FFPE tissue kit (QIAGEN) was used to extract genomic DNAs from FFPE samples. DNA concentration was determined using spectrophotometry (NanoDrop 2000C; Thermo Scientific, Wilmington, DE) and adjusted to 10 ng/µl. The criterion for DNA purity was optical density (OD) _260_/OD_280_ ≥ 1.8. For quantitative PCR (qPCR) to determine gene copy number, double-strand DNA quantification kit (Quant-iT™ PicoGreen dsDNA Assay kit, Invitrogen, Carlsbad, CA) was used to quantify DNA concentration, and DNA concentration was adjusted to 1 ng/µl. Total RNAs were extracted from surgically resected fresh-frozen tissues and cells isolated from body cavity fluids with RNeasy Mini kit (QIAGEN) following standard protocols, and quantified by spectrophotometry (NanoDrop 2000C; Thermo Scientific), and RNAs indicating OD_260_/OD_280_ ≥ 1.8 were used for detection of fusion genes.

*Pyrosequencing for detection of single-nucleotide variations*

Pyrosequencing was used for detection of single-nucleotide variations (SNVs) in eight genes (*EGFR*, *KRAS*, *BRAF*, *PIK3CA*, *NRAS*, *MEK1*, *AKT1* and *PTEN*). An internal fragment of each gene was amplified by PCR with the PyroMark PCR Kit (QIAGEN) using 20 ng of genomic DNA and primers specific for each genomic region. PCR products were sequenced on the PyroMark Q24 pyrosequencer (QIAGEN) using PyroMark Gold Q96 Reagents (QIAGEN) and sequencing primers specific for each genomic region.

*Fragment size analysis for detection of insertion/deletion-type genetic alterations*

Three insertion/deletion-type genetic alterations in *EGFR* and *HER2* were determined by sizing PCR-amplified products using capillary electrophoresis (QIAxcel Advanced System; QIAGEN) with the QIAxcel DNA High Resolution Kit (QIAGEN). PCR was performed with 20 ng of genomic DNA, primers specific for each genomic region and PyroMark PCR Kit (QIAGEN).

*Copy number analysis*

qPCR with SYBR green for evaluation of amplification of five genes (*EGFR*, *MET*, *PIK3CA*, *FGFR1* and *FGFR2*) was performed on the StepOnePlus^TM^ Real time PCR system (Applied Biosystems, Foster City, CA) using 2 ng genomic DNA, PCR primers for each gene and SYBR^®^ Premix Ex Taq™ II (Tli RNaseH Plus) (TAKARA BIO, Shiga, Japan). To quantify target gene copies, standard calibration curves were generated using serial dilutions (10^2^–10^8^ copies) of recombinant plasmid DNA for each gene using plasmids constructed in the pCR2.1-TOPO vector (Invitrogen). The copy number of each gene was normalized using the copy number of *LINE-1*. Gene copy number changes were determined by the ratio of the normalized quantity of the target gene to that of *COL8A1*. Results that were $\geq$2-fold higher than the average value in negative control cell lines and human genome DNAs (Clontech, Palo Alto, CA; Promega, Madison, WI) were considered to be gene amplification. DNAs extracted from following cell lines with copy-number gain in each gene were used for the assay positive control; *EGFR* (HCC827 [1], A431 [2]), *MET* (EBC-1 [1], NCI-H2170 [1]), *PIK3CA* (Calu3 [3], NCI-H520 [4]), *FGFR1* (Calu3 [5], NCI-H1703 [5]) and *FGFR2* (SNU-16 [6], KATOIII [6]).

*Detection of fusion genes*

*ALK*, *ROS1*, and *RET* fusions were detected by reverse-transcription PCR (RT-PCR) using RNA from fresh-frozen samples and cells isolated from body cavity fluids. Synthesis of cDNA templates was performed with total RNA (1 μg), Random Primer (hexadeoxyribonucleotide mixture; pd(N)_6_) (TAKARA BIO) and Omniscript RT Kit (QIAGEN). RT–PCR reactions the expression of *GAPDH* was used as a positive control. Detection of *EML4-ALK* and *ROS1* fusion genes (*CD74-ROS1* and *SLC34A2-ROS1*) were performed according to those developed by Sun *et al.* [7] and Li *et al.* [8], respectively. Information on the primers and methods for detecting *KIF5B-RET* and *CCDC6-RET* fusion genes were kindly provided by Dr. Takashi Kohno (National Cancer Center, Tokyo, Japan). Additionally, tumor samples that were positive for *ALK* fusions were also screened by immunohistochemistry (IHC) using the intercalated antibody-enhanced polymer method [9] with Anti-ALK antibody (5A4) (Abcam, Cambridge, MA), polyclonal Rabbit Anti-Mouse Immunogloblins (Dako, Carpinteria, CA) and Envision + Kit Rabbit (Dako), and confirmed by break-apart fluorescence in situ hybridization (FISH) using the Vysis ALK Break Apart FISH probe kit (AbbottMolecular, Des Plaines, IL).

1. ***EGFR* mutation tests by the commercial clinical laboratory.**

*DNA extraction methods*

Xylene treatment to remove paraffin from FFPE sections, followed by use of the QIAamp DNA Mini Kit (QIAGEN) according to the manufacturer’s instructions. Fluid cytology samples were loaded four times on to the same column to ensure all DNA was extracted.

*Scorpion ARMS*

The 20 μL DNA admixture samples (10 ng/μL) provided for ARMS analysis were diluted with 70 μL water, giving the highest DNA input/assay (40 μL sample required for all assays in the DxS ARMS kit) while providing sufficient sample volume for confirmatory analysis. After dilution, the samples were quantified using RNAase P. The 22 μL aliquots of DNA extracted from the BB samples were quantified using Quant-IT PicoGreen dsDNA reagent (Invitrogen Inc.). All samples with a DNA concentration >1 ng/μL were diluted 1:1 with water to give a total of 44 μL. Any samples still >4 ng/μL were further diluted 1:1. Any sample at a DNA concentration <1 ng/μL was not diluted and tested for exon 19 deletions, L858R, and T790M mutations only.

*EGFR* ARMS assays were performed once for each sample, in the first instance, as directed in the DxS 29 Mutation kit pack leaflet with the following modifications. Modified cut-off values (delta Ct values, dCt) were used to define mutation-positive samples: dCt was 8 for T790M, 9 for exon 19 deletions, and 11 for L858R, L861Q, G719X, S768I, and insertions. Modified analysis instructions for the T790M assay were generated and agreed with DxS in order to avoid the reporting of false positive results: analyses for T790M were repeated in triplicate for samples with a dCt of ≥8 or a T790M Ct ≥33 and samples classed as mutation-positive if all three replicates had a dCt of ≤8. For any sample with an indeterminate result (a dCt >7 but below the cut-off point where applicable), for any of the mutations, the analysis was repeated in triplicate and only if all three replicates gave a dCt value in the positive zone was the sample classed as mutation positive. Any mutation-negative sample that had a control Ct >35 was classified as an analysis failure (insufficient material present to confidently assign a mutation status).

*Cycleave method*

This assay was performed using Cycleave PCR core kits (TaKaRa Bio Inc.) using AmpliTaq Gold^®^ DNA polymerase (Applied Biosystems) as previously described [10], and primers and probes for L858R, T790M, G719(S/A/C), and L861Q (TaKaRa Bio Inc.).

Exon 19 deletions were determined by common fragment analysis using PCR with a FAM-labeled primer set [10], and the products electrophoresed on an ABI3130 Genetic Analyzer (Applied Biosystems) and Gene Mapper Software used to detect exon 19 deletions.

**REFERENCES**

1. McDermott U, Sharma SV, Dowell L, Greninger P, Montagut C, Lamb J, et al. Identification of genotype-correlated sensitivity to selective kinase inhibitors by using high-throughput tumor cell line profiling. Proc Natl Acad Sci U S A. 2007;104: 19936-19941.

2. Moroni M, Veronese S, Benvenuti S, Marrapese G, Sartore-Bianchi A, Di Nicolantonio F, et al. Gene copy number for epidermal growth factor receptor (EGFR) and clinical response to antiEGFR treatment in colorectal cancer: a cohort study. Lancet Oncol. 2005;6: 279-286.

3. Yamamoto H, Shigematsu H, Nomura M, Lockwood WW, Sato M, Okumura N, et al. PIK3CA mutations and copy number gains in human lung cancers. Cancer Res. 2008;68: 6913-6921.

4. Spoerke JM, O'Brien C, Huw L, Koeppen H, Fridlyand J, Brachmann RK, et al. Phosphoinositide 3-kinase (PI3K) pathway alterations are associated with histologic subtypes and are predictive of sensitivity to PI3K inhibitors in lung cancer preclinical models. Clin Cancer Res. 2012;18: 6771-6783.

5. Dutt A, Ramos AH, Hammerman PS, Mermel C, Cho J, Sharifnia T, et al. Inhibitor-sensitive FGFR1 amplification in human non-small cell lung cancer. PLoS One. 2011;6: e20351.

6. Matsumoto K, Arao T, Hamaguchi T, Shimada Y, Kato K, Oda I, et al. FGFR2 gene amplification and clinicopathological features in gastric cancer. Br J Cancer. 2012;106: 727-732.

7. Sun Y, Ren Y, Fang Z, Li C, Fang R, Gao B, et al. Lung adenocarcinoma from East Asian never-smokers is a disease largely defined by targetable oncogenic mutant kinases. J Clin Oncol. 2010;28: 4616-4620.

8. Li C, Fang R, Sun Y, Han X, Li F, Gao B, et al. Spectrum of oncogenic driver mutations in lung adenocarcinomas from East Asian never smokers. PLoS One. 2011;6: e28204.

9. Takeuchi K, Choi YL, Togashi Y, Soda M, Hatano S, Inamura K, et al. KIF5B-ALK, a novel fusion oncokinase identified by an immunohistochemistry-based diagnostic system for ALK-positive lung cancer. Clin Cancer Res. 2009;15: 3143-3149.

10. Yatabe Y, Hida T, Horio Y, Kosaka T, Takahashi T, Mitsudomi T. A rapid, sensitive assay to detect EGFR mutation in small biopsy specimens from lung cancer. J Mol Diagn 2006; 8: 335–341.
